# Simultaneous visualization of extrinsic and intrinsic axon collaterals in Golgi-like detail for mouse corticothalamic and corticocortical cells: a double viral infection method

**DOI:** 10.3389/fncir.2014.00110

**Published:** 2014-09-17

**Authors:** Akiya Watakabe, Masafumi Takaji, Shigeki Kato, Kazuto Kobayashi, Hiroaki Mizukami, Keiya Ozawa, Sonoko Ohsawa, Ryosuke Matsui, Dai Watanabe, Tetsuo Yamamori

**Affiliations:** ^1^Division of Brain Biology, National Institute for Basic BiologyOkazaki, Japan; ^2^Department of Molecular Genetics, Institute of Biomedical Sciences, Fukushima Medical University School of MedicineFukushima, Japan; ^3^Division of Genetic Therapeutics, Center for Molecular Medicine, Jichi Medical UniversityShimotsuke, Japan; ^4^Department of Molecular and Systems Biology, Graduate School of Biostudies, Kyoto UniversityKyoto, Japan

**Keywords:** retrograde viral vector, AAV, axon collateral, corticothalamic, pyramidal, tracer

## Abstract

Here we present a novel tracing technique to stain projection neurons in Golgi-like detail by double viral infection. We used retrograde lentiviral vectors and adeno-associated viral vectors (AAV) to drive “TET-ON/TET-OFF system” in neurons connecting two regions. Using this method, we successfully labeled the corticothalamic (CT) cells of the mouse somatosensory barrel field (S1BF) and motor cortex (M1) in their entirety. We also labeled contra- and ipsilaterally-projecting corticocortical (CC) cells of M1 by targeting contralateral M1 or ipsilateral S1 for retrograde infection. The strength of this method is that we can observe the morphology of specific projection neuron subtypes en masse. We found that the group of CT cells extends their dendrites and intrinsic axons extensively below but not within the thalamorecipient layer in both S1BF and M1, suggesting that the primary target of this cell type is not layer 4. We also found that both ipsi- and contralateral targeting CC cells in M1 commonly exhibit widespread collateral extensions to contralateral M1 (layers 1–6), bilateral S1 and S2 (layers 1, 5 and 6), perirhinal cortex (layers 1, 2/3, 5, and 6), striatum and claustrum. These findings not only strengthened the previous findings of single cell tracings but also extended them by enabling cross-area comparison of CT cells or comparison of CC cells of two different labeling.

## Introduction

Classification of neuronal type is prerequisite to the understanding of cortical circuit. Although genetic identification of cell types using transgenic mouse lines is rapidly becoming ever more sophisticated (Fishell and Heintz, 2013; Huang and Zeng, 2013), identification by projection target is still one of the most reliable criteria for cell type classification of the cortical neurons that is applicable across species (Nelson et al., 2006; Molyneaux et al., 2007; Thomson and Lamy, 2007; Sorensen et al., 2013). One caveat of identification by projection target, however, is that cortical neurons often have axon collaterals that project to multiple targets (Thomson and Lamy, 2007; Rockland, 2013). To fully characterize the “cell type,” there is thus a need to analyze axon collateral projections. Investigation of axon collaterals is also important in understanding the cortical microcircuitry (Thomson and Lamy, 2007; Morishima et al., 2011). At present, however, our knowledge on extrinsic and intrinsic collaterals is still limited mainly due to technical difficulty.

In past studies, the distribution of axon collaterals has been visualized by labeling sparsely only a few cells *in vivo* or *in vitro* and tracing projections from each cell in their entirety (Deschenes et al., 1994; Bourassa et al., 1995; Zhang and Deschenes, 1997; Briggs and Callaway, 2001; Morishima et al., 2011; Kaneko, 2013). Such reconstructions are highly laborious and technically demanding. Potential alternative approach is the use of retrograde viral vectors (Wickersham et al., 2007; Kato et al., 2013). By incorporating “TET-Off system” to lentiviral-based retrograde vector, we previously showed that we can visualize the fine morphology of specific projection neuron subtypes (Watakabe et al., 2012). Unfortunately, simple retrograde approach was not suited for the analyses of axon collaterals, because the infected cells often spread widely across various brain regions. To analyze the complex network of collateral projections, we needed a method that can restrict the infection to a more limited population of cells. To achieve this goal of more specific labeling, we separated the two components of TET-Off system, namely, the tetracycline transactivator (tTA) under the cellular promoter and a transgene under the tetracycline responsive element (TRE), into retrograde and locally infecting viral vectors. We reasoned that by injecting these vectors into one prospective target structure and a known origin of these connections, high-level transgene expression would occur only in the doubly infected neurons. If this strategy works successfully, we can expect to label specific projection cell types in their entirety, including both the intrinsic and extrinsic axon collateral branches.

In this paper, we used this double infection strategy to characterize CT cells in the somatosensory barrel field (S1BF) and motor cortex (M1) and CC cells projecting to contralateral M1 or ipsilateral S1 of mice. Our data demonstrated that CT and CC cells both send extensive axon collaterals to multiple extrinsic targets. They were, however, distinct in their collateralization patterns. We also found that layer-specific distribution of intrinsic collaterals of CT cells is conserved across areas. Developmentally, “subcerebral-projecting neurons” and “callosal-projecting neurons” are fate-determined in the early phase of cortical development (Koester and O'Leary, 1993; Britanova et al., 2008; Leone et al., 2008). We suggest that such developmental background may be reflected in the distinctive collateralization patterns of CT and CC cells in adults. Our data not only strengthen the morphological understandings of these cell types obtained in the past single cell tracing studies but also extend them by providing a simple means for cross-area comparison or comparison of differential labeling.

## Materials and methods

### Ethics statement

All the experiments were conducted in accordance with the guidelines of the National Institutes of Health, and the Ministry of Education, Culture, Sports, Science, and Technology (MEXT) of Japan, and were approved by the Institutional Animal Care and Use Committee of National Institutes of Natural Sciences. We made all efforts to minimize the number of animals used and their suffering. AAV and lentiviral vectors were handled as Biosafety Level 1 (BSL-1) and BSL-2 materials, respectively. All the viral injection experiments were approved by the Recombinant DNA committee of National Institute for Basic Biology.

### Plasmid construction

**Figure 1:**
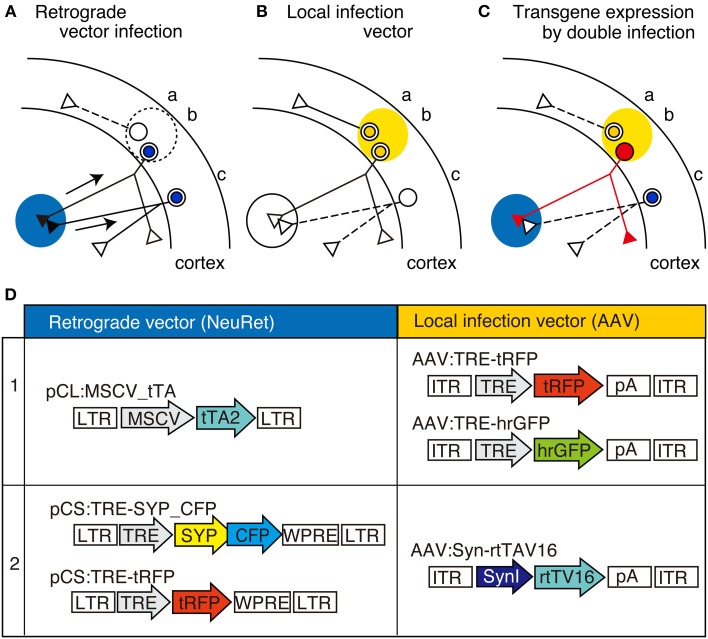
**The schematic view of TET double infection strategy and the viral vector constructs used for the experiments. (A–C)** Schematic view of our double infection method, which utilizes TET system. In this scheme, AAV is injected into the cortex (shown by yellow shade) as the local infection vector, while the NeuRet vector is injected into the subcortical region (shown by blue shade) as the retrograde vector. These vectors contain either tTA or TRE-transgene as depicted in **(D)**. In these panels, three neurons indicated as “a,” “b,” and “c” are shown. In panel **(A)**, the neurons infected with the retrograde vector (“b” and “c”) are indicated by blue nuclei. The arrows represent the retrograde transport of the viral particles. In **(B)**, the neurons infected with the local infection vector (“a” and “b”) are indicated by yellow nuclei. Panel **(C)** shows the consequence of double infection. Since the tTA and TRE-transgene are both present in neuron “b,” high-level expression of transgene takes place to fill the entire neuron with the transgene product (represented by red color). No transgene expression occurs when the neurons are infected by either of the two viral vectors (e.g., neurons “a” and “c”). **(D)** Schematic representations of the viral constructs we used in this study. CFP, celulean; SynI, human synapsin I promoter; SYP, synaptophysin; tTA2, “TET-Off” tetracyclin transactivator; rtTV16, “TET-ON” reverse tetracyclin transactivator; tRFP, turboFP635; LTR, long terminal repeat; ITR, inverted terminal repeat; TRE, tetracycline responsive element; pA, poly(A) site; WPRE, woodchuck hepatitis virus post-transcriptional regulatory element.

The constructs used in this study are schematically shown in Figure 1. The plasmid pCL20c:MSCV_tTA was constructed by replacing the GFP sequence of pCL20c:MSCV_GFP (Kato et al., 2007) with tTA2, TET-Off activator. The plasmid pCS:TRE-tRFP was constructed by subcloning TurboFP635 (tRFP) (Everogen) and WPRE (woodchuck hepatitis virus posttranscriptional regulatory element), downstream of TRE promoter in pCS vector (Miyoshi et al., 1998). The plasmid pCS:SYP_CFP was constructed by replacing the tRFP sequence of pCS:TRE-tRFP with the fusion protein of synaptophysin and CFP. For this construct, synaptophysin gene fragment was amplified by PCR using primers, 5′-gcGCTAGCgccaccATGGACGTGGTGAATCAGCT-3′ and 5′-caactcgagCTGATTGGAGAAGGAGGTGG-3′, and cloned in frame with CFP (Cerulean; courtesy of Dr. David Piston). AAV:TRE-tRFP was constructed by subcloning the DNA fragments containing TRE promoter and tRFP gene between *Mlu*I and *Bgl*II sites of pAAV-MCS (Agilent Technologies). AAV:SynI_rtTV16 was constructed by subcloning human synapsin I promoter (Hioki et al., 2009) and rtTV16, a variant of reverse tetracycline transactivator (Kinoshita et al., 2012) between *Mlu*I and *Bgl*II sites of pAAV-MCS.

### Viral injection

The lentiviral preparations used for *in vivo* injection experiments were produced in large scale and purified by ion exchange column chromatography as previously described (Kato et al., 2011c). The titers of the lentiviral preparations were measured by RT-PCR. They were in the range of 1.0–3.0 × 10e12 copies/ml. For this experiment, a lentiviral vector pseudotyped with fusion glycoprotein C type (FuG-C) for neuron-specific retrograde gene transfer (NeuRet) was used as the retrograde vector (Kato et al., 2011c). Adeno-associated viral (AAV) vectors used in this paper have ITRs of AAV2 and with capsids of serotype 1. They were produced in HEK 293 cells by using a helper virus free system and purified by two times CsCl2 density gradients and titrated by Q-PCR as described previously (Yagi et al., 2011). Final preparations were dialyzed against PBS and adjusted to 1.0 × 10e12 vg/ml. To prevent adhesion of the AAV vector to the glass micropipettes, 0.001% Pluronic-F68 Solution (Sigma-Aldrich) was added to the vector solution.

Eighteen adult C57BL6J mice of either sex (17–28 weeks) were anesthetized with an intraperitoneal injection of ketamine/xylazine mixture (100, 10 mg/kg, respectively). A small hole was made in the skull using a dental drill. The viral vector was delivered by pressure injection using a glass micropipette (tip size of roughly 40 μm for cortical injection and 50–70 μm for subcortical injection) attached to a Nanoliter 2000 injector connected to Micro4 controller (World Precision instrument). To inject virus into the brain, the dura was punctured using a tip of 27G needle, through which the glass pipette was slowly lowered to the target depth. Approximately 0.5 μl (for cortical injections) or 0.4–1 μl (for subcortical injections) of the viral solutions were injected at the rate of 0.1–0.2 μl/min. For co-injection experiment with the fluorescent tracer, the viral solution was mixed with one-fifth volume of Cholera Toxin Subunit B-Alexa Fluor 488 conjugate (CTB-Alexa488; final 0.2 mg/ml, Invitrogen, #C-22841). The pipette was held in place for 2 min before and after the injection. After retracting the glass micropipette, the hole was filled with Spongel, an absorbable gelatin sponge (Astellas Pharma Inc.) and the head skin was sutured. The mice were sacrificed 3–4 weeks after injection. For those injected with “TET-ON” vectors, doxycycline was supplied from the drinking water (2 mg/ml).

### Immunostaining and data analyses

Mice were anesthetized with sodium pentobarbital and perfused transcardially with 0.9% NaCl, followed by fixation with 4% paraformaldehyde in 0.1 M phosphate buffer, pH 7.4. For double or triple immunofluorescence, the sections were treated with 80% methanol/20% dimethyl sulfoxide solution (Dent's solution) for more than 30 min and digested with proteinase K (0.2 μg/ml) at 4°C for 12 h. The proteinase K treatment was omitted for vGluT2 staining. After washing, the sections were blocked with 10% fetal bovine serum, 2% bovine serum albumin, and 0.5% Triton X100 in TBS, pH7.4, followed by overnight incubation with the primary antibody for GFP (1:1000; chick polyclonal, abcam, ab13970) and/or for tRFP (1:5000; rabbit polyclonal, Evrogen, #AB233) and/or for vGluT2 (1 μg/ml, guinea pig polyclonal; Kaneko et al., 2002) at 4°C. After incubation with the Cy3-conjugated (anti-rabbit Cy3, 1:2000; Jackson ImmunoResearch), and/or AlexaFluor 488-conjugated (anti-chicken, 1:2000, Invitrogen, #1008651) and/or Cy5-conjugated (anti-guinea pig Cy5, 1:2000; Jackson ImmunoResearch) secondary antibodies, the sections were counterstained with Hoechst 33342 (1:2000; Molecular Probes). The fluorescent images were captured by Olympus DP71 digital camera attached to BX51 microscope (Olympus). The confocal images were taken by Nikon confocal laser microscope system A1. Maximum intensity projection images for the confocal data were created by NIS-Elements imaging software (Nikon). The detailed information about the images used in the figures are listed in Table S1. All the images were processed by Adobe photoshop for proper contrast for presentation. At high magnification, we were able to observe small protrusions coming out of neurite processes, which often shaped like dots on sticks. They were positively identified as the spines when they were coming out of thick processes, which we identified as dendrites. We also encountered thin fibers that have dots on them, which we identified as the axons and en-passant boutons, respectively. The annotations for axons and dendrites in the figures were determined by these criteria.

To analyze the lamina profiles for CT cells, the sections were immunostained with anti-tRFP, anti-GFP, and anti-vGluT2 antibodies and counterstained with Hoechst 33342 as above and the fluorescent images in four channels were obtained by confocal microscopy. For the analyses of S1BF, data of fluorescence intensity for seven hemispheres (six mice) were averaged after normalization as follows. First, a vertical strip of 310 μm width was cut out from the photos of the stained sections. In case the dendrites did not run vertical to the surface, the image was “transformed” in Photoshop (Adobe) so that the direction of the column is set vertical to the layer. The pia to the white matter portion was cut out from this strip and the size was adjusted to 300 × 100 pixel rectangle. The lamina plots of fluorescence intensity in four channels were measured using “Plot Profile” menu of ImageJ (http://imagej.nih.gov/ij/). The value for each plot was normalized against the sum of all the 300 plot values after subtracting the averaged background. To determine the lamina plots for the cell bodies, the contrast of the original data was adjusted so that only the cell bodies and thick fibers remain. The contrast-adjusted image was further converted to a binary image in ImageJ using “erode” and “dilate” menu to get rid of small dots that are not cell bodies. Lamina plot for this image was made in the same way as other images except that the value was made 1/10 for the purpose of viewing. For the analyses of M1, data of fluorescence intensity for four hemispheres (four mice) were averaged essentially the same way as shown above for S1BF but with some modifications. Because the columnar structure of M1 is expanded toward the pia matter, we selected the region of interest (ROI) as a fan-shaped trapezoid so that the neurons in a “column” are all included in ROI. The intensity of each 300 lamina point was first normalized against the “area” and then processed similarly as for S1BF.

To quantify the distribution of collateral terminals of corticocortical labeling (mouse #442, 456 and 457), we immunostained the tissue sections with the anti-GFP antibody to visualize the SYP-CFP signals and retrieved the fluorescent images by DP71 digital camera in exactly the same condition (ISO, exposure time etc.) for the same mouse samples. We set a Region Of Interest (ROI) for measurement at the most densely innervated portion of the image. In the cerebral cortex, the ROIs were selected so that they span all layers. The raw image of the fluorescent signals were converted to an 8 bit gray image and binarized at multiple threshold values (10, 20, 30, 40, and 50) for signal segmentation. The “area fraction” of the positive pixels within each ROI was then measured for these threshold values by ImageJ software (Figure S3). As Figure S3B shows, the signal density thus measured were greatly affected by the threshold values. However, the signal density values of various brain regions normalized against ipsilateral S1 were relatively constant irrespective of threshold (Figure S3E), except at Th10 (Figure S3D). The graphs in **Figure 8J** and Figure S3G were thus made using the signal densities at TH30.

## Results

### CT cells were efficiently labeled by TET double infection method

Figures 1A–C show the principle of our double infection strategy, which hereafter we refer as TET double infection method. This method requires the injection of two types of viral vectors. One is the “NeuRet” vector, pseudotyped with FuG-C, a chimeric glycoprotein composed of parts of rabies virus glycoprotein and vesicular stomatitis virus glycoprotein, which exhibits enhanced ability for retrograde gene transfer (Figure 1A; Kato et al., 2011a,b,c). The other is the adeno-associated viral vector (AAV) for local infection (Figure 1B). The point of the method is to supply tTA and TRE-transgene separately using these vectors (Figure 1D) so that high-level expression of the transgene occurs only when the cells are doubly infected (Figure 1C, red cell “b”).

**Figure 2:**
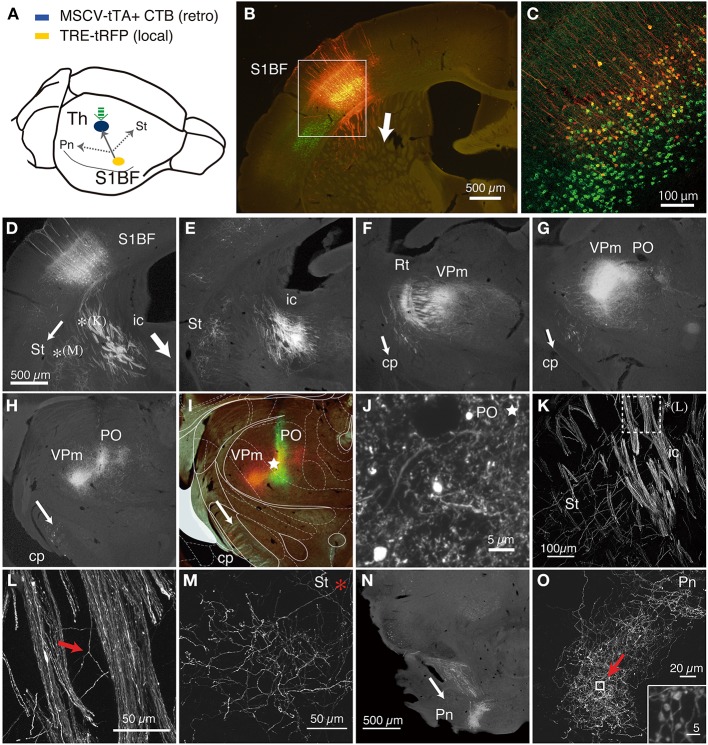
**CT cells were efficiently labeled by TET double infection method. (A)** Schematic view of double injection. The NeuRet vector encoding tTA (MSCV_tTA) was injected into the thalamus (shown by green arrow) as a retrograde vector, together with CTB-Alexa488. The AAV vector carrying TRE-tRFP was injected into S1BF. We observed collateral projections to the thalamus (Th), striatum (St) and pons (Pn). **(B)** The coronal section at the level of S1BF showing CT cells that were labeled as red by expression of tRFP. The green signals are also CT cells retrogradely labeled by CTB-Alexa488. The arrow indicates the direction of corticothalamic projections. **(C)** Magnified view of the white square in **(B)**. The contrast of this confocal image is adjusted so that we can identify each labeled cell body. **(D–I)** The coronal images of the tRFP signals that follow the one in **(B)** were aligned in order from anterior to posterior. The arrows indicate the directions of the collateral projections labeled by this strategy. **(D)** The striatal collateral splits at the level of **(D)** [^*^(K) and ^*^(M); higher magnification views in **(K,M)**]. In **(E–H)**, the thalamic projection that proceeded in the internal capsule (ic) innervate reticular thalamic nucleus (Rt), VPm and PO, while the split collaterals proceed within the cerebral peduncle (cp). **(I)** Multicolor merged view for **(H)**, which is just around the thalamic injection site. The green signals indicate the local deposit of CTB-Alexa 488, which mark the injection center for the retrograde vector. The asterisk shows the position of **(J)**. The Paxinos atlas (Paxinos and Franklin, 2003) was superimposed to the image in **(H)** to make **(I)**, to show the identification of the spots of concentrated tRFP signals. **(J)** A magnified view of the injection site indicated by asterisks in **(I)**. Note that we can examine the fine branches with boutons even around the injection site. A higher magnification view is presented in Figure S2. **(K)** A magnified view of the striatal collaterals that branched out of the CT bundles of internal capsule (see **D** for low magnification view). The dotted square [denoted as ^*^(L)] is magnified in **(L)** to indicate the example of collateral branching (indicated by red arrow). **(M)** A magnified view of the striatal collaterals that arborized at the final destination (see **D** for low magnification view). **(N)** A low power view of the pontine collaterals. **(O)** A magnified view of the terminal arborization of pontine collaterals. The boxed region indicated by a red arrow is magnified in the inset, which exhibits a cluster of large boutons. The images used in **(C,J–O)** are maximal projection stacks of confocal sections. S1BF, somatosensory barrel field; Th, thalamus; Pn, pons; St, striatum; ic, internal capsule; cp, cerebral peduncle; Rt, reticular thalamic nucleus; VPm, ventral posteromedial nucleus; PO, posterior thalamic nuclear group.

To test the validity of this idea, we injected a NeuRet vector encoding tTA (tTA2; TET-Off) under the ubiquitous MSCV promoter, and an AAV vector encoding red fluorescent protein, tRFP, under the TRE promoter (combination 1 of Figure 1D), into the mouse thalamus and somatosensory barrel field (S1BF), respectively, and waited for 3–4 weeks of expression (Figure 2A). In this and following experiments, the expression of the fluorescent proteins were detected by immunofluorescence, so that we can efficiently detect both min structures such as spines and boutons, as well as main dendrites and cell bodies, which exhibited strong signals without antibody detection.

As we had expected, the double viral injections resulted in robust expression of tRFP in the deep layers of S1BF (Figure 2B). Two lines of evidence indicate that these are authentic signals of the CT cells doubly infected by the NeuRet and AAV vectors. First, in the control experiment in which we omitted thalamic injection of the retrograde vector, AAV: TRE-transgene (hrGFP) failed to produce expression above background level: there existed basal activity of TRE promoter, but the diffuse and faint staining was only observed after immunostaining and restricted to the main shafts and soma of some cells (Figure S1). Second, we had injected a fluorescent retrograde tracer, CTB-Alexa488, together with the NeuRet vector in Figure 2. The tRFP expression was observed only in the CTB-positive cells (Figure 2C), suggesting that the infection occurred in a retrograde manner. Whereas CTB uniformly labeled layer 6 neurons, the tRFP-positive cells preferentially located in the upper part of layer 6. Considering the differential sublamina localization of VPm and POm-targeting CT cells (Killackey and Sherman, 2003), it is possible that the NeuRet vector spread only within VPm, while CTB spread both VPm and POm. The percentages of the tRFP-positive cells among the CTB-positive cells varied across experiments and across regions of interest, but were 31–51% in the 100 × 100 μm core of the labeling (*n* = 3, data not shown). The absence of tRFP signals above layer 4 also indicates that TRE-tRFP vector alone cannot produce strong fluorescent signals. What was most convincing, however, was that we could actually follow the trajectory of the neurons from the cortex to the thalamus (Figures 2D–H).

This series of panels starts at the level of injection (Figures 2B,D), in which the main tracts vertically descended down to the white matter and either penetrated into the striatum or turned perpendicular along the white matter to the ventral side (the directions of axonal projections are represented by white arrows). In either case, the tRFP-positive axons eventually entered the striatum to be a part of the internal capsule (ic). At higher magnification, we were able to identify the individual axons that constitute the thick bundles of the internal capsule (Figures 2K,L). These axons reached ventral posteromedial nucleus (VPm) and posterior thalamic nuclear group (PO), the subnulei of the thalamus (Figures 2H,I), by way of the reticular nucleus (Figure 2F; Rt), where they branched off into fine terminals. It is a big advantage of the double infection method that we can investigate the terminal morphology even around the injection site (Figure 2J, asterisk, Figure S2). At higher magnification, we could observe fine boutons and axons except in the very vicinity of injection site. Given the volume of injection (0.4 μl), we believe that CTB-Alexa488 and NeuRet vector spread more widely than it remained as a green deposit after several weeks of waiting period, thereby taken up by diverse terminals in the thalamus.

Regarding the toxicity of the vector systems, we observed only a minor damage to the double infected cells (Figure S1). We observed loss of neuronal cell bodies just around the injection site (100–200 μm, Figure S1I), which is probably due to physical damage of pressure injection as well as high concentration of vector preparation. Outside this damaged zone, however, we observed normal NeuN signals even for neurons with high transgene expression (Figure S1K).

In addition to this main tract, we could observe two other collateral branching. First, we observed corticostriatal collaterals to split from the main tract within the striatum and run parallel to the white matter [Figures 2D,^*^(K),K,L], until they reached the lateral side of the striatum for innervation (Figure 2M). Second, a group of axons separated from the main tract before entering the reticular nucleus (Figure 2F) and proceeded within the cerebral peduncle (cp) toward pons and other midbrain structures (Figures 2G,H). They branched off into numerous fine terminals at the level of pons (Figures 2N,O), where they formed large (2–3 μm) boutons (Figure 2O, inset). Based on previous single cell tracing studies (Deschenes et al., 1994; Bourassa et al., 1995; Levesque et al., 1996), these collaterals are considered to stem from the layer 5 “corticothalamic” cells. Relatively low number of axons that pass the cerebral peduncle is consistent with the number of layer 5 cells that were labeled in this experiment (e.g., only two cells in Figure 2C).

### CT cell terminal morphology can be visualized by TET double infection method

One advantage of TET double infection method is the versatility of combinations for the local and retrograde infection vectors. In the next series of experiments, we tested several new options. First, we exchanged the combination for tTA and TRE vectors so that TET activator is expressed from the local vector and TRE is supplied from the retrograde vector (combination 2 of Figure 1D). This time, “TET-On” system was used instead of “TET-Off”: a modified reverse tetracycline transactivator, rtTV16 (Kinoshita et al., 2012) under synapsin promoter (Hioki et al., 2009), was expressed from AAV in S1BF. As the TRE-transgene, we constructed a NeuRet vector encoding the fusion protein of synaptophysin (SYP) and CFP, which is expected to serve as the marker for the presynaptic terminal structure (e.g., Li et al., 2010). We injected this SYP_CFP vector together with the tRFP vector into the thalamus, so that we can visualize both the axon fibers and terminations. After several weeks of waiting period with doxycycline supplied from the drinking water, the mouse was sacrificed for histological processing.

**Figure 3:**
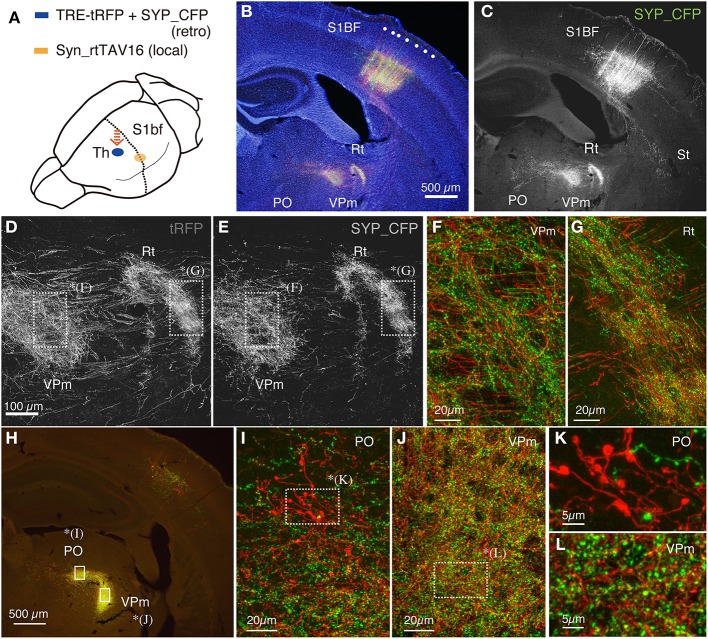
**Terminal bouton structures of CT cells visualized around the injection site**. Two retrograde vectors, carrying TRE-SYP_CFP and TRE-tRFP were mixed and injected into the thalamus and AAV vector carrying Syn-rtTAV16 was injected into S1BF. The expressed SYP_CFP (green) and tRFP (red) were detected by immunostaining. SYP_CFP signals were generally more granular with weaker staining in the axonal fibers compared with the tRFP signals. **(A)** Schematic view of double injection. **(B)** The coronal section at the level of S1BF showing the soma and thalamic terminations of CT cells. The injection sites are caudally shifted from those shown in Figure 2. Counterstaining with Hoechst nuclear dye revealed the area and lamina structure of the cortex (blue). Each white dot represents barrel structure. Both SYP_CFP and tRFP signals are shown in green and red, respectively. **(C)** Only SYP_CFP signals are shown in gray. Note that SYP_CFP fusion protein exhibits less axon fiber staining than tRFP. **(D,E)** Magnified views of VPm and reticular thalamic nucleus stained for tRFP and SYP_CFP. **(F,G)** Overlay of SYP_CFP (green) and tRFP (red) signals in VPm and PO. Axonal fibers were better visualized by tRFP, while granular staining was enhanced for SYP_CFP. **(H)** The coronal section approximately 0.8 mm caudal to **(B,C)**. At this level, the CT terminations in VPm and PO are well segregated. **(I,J)** Magnified views of PO and VPm of **(H)**. **(K,L)** Further magnified views of PO and VPm from **(I,J)**, respectively. Note clusters of large boutons in PO as compared to the small ones in VPm. The images used in **(D–G,I–L)** are maximal projection stacks of confocal sections.

As shown in Figure 3, the new combination of local/retrograde infection vectors worked as successfully as the previous combination. Since we shifted the site of cortical injection caudally, the soma and the terminations of the CT cells were visible in the same section (Figures 3B,C). At high magnification, both SYP_CFP and tRFP signals exhibited granular distributions in the terminal regions in the reticular nucleus and VPm (Figures 3D–G). Although not many cells were double labeled by tRFP and SYP_CFP in this condition, the tRFP-positive granules were co-labeled with SYP_CFP in the double-labeled granules (data not shown), suggesting that they represent presynaptic boutons. Note that SYP_CFP fusion protein exhibits less fiber-like staining than tRFP. The presynaptic localization of SYP_CFP was, however, not absolute. As shown in Figures 3E–G, SYP_CFP signal (green) was present at low level in the axonal fibers as well. Conversely, synaptic boutons were visualized by tRFP expression as granular staining attached to the axonal fibers. Thus, both these fluorescent reporters can be used to label the terminal structures, albeit at a different preference for fiber/terminal localization. We also note here that we observed background staining of cell soma by retrograde infection of TRE vector alone, which is considered to occur with the basal activity of the TRE promoter. Staining was weak and diffuse, and was clearly different from the double infected cells. We also found, in rare instances, strongly positive cells in unexpected locations. This could be either contamination during injection or retrograde infection of AAV1 (Burger et al., 2004).

It is previously reported that there are two kinds of terminal endings from CT cells of layers 5 and 6: whereas layer 6 CT cells have small endings (<1 μm) in VPm and PO, layer 5 CT cells have giant endings (2–10 μm) in PO only (Bourassa et al., 1995; Rouiller and Welker, 2000). To test whether we can also observe two types of endings, we examined the terminal morphology of CT cells in VPm and PO by confocal microscopy at high resolution. These two thalamic regions exhibited dense staining for tRFP and SYP_CFP at low resolution (Figure 3H) and contained numerous granules at high resolution (Figures 3I–L). Consistent with the previous reports, we observed clusters of giant endings in PO (Figures 3I,K) but not in VPm (Figures 3J,L). Thus, fine terminal morphology can be delineated even around the injection site without interference, which is not possible in the conventional tracer experiments.

### CT cells in S1BF have highly layer-specific axonal/dendritic fields

**Figure 4:**
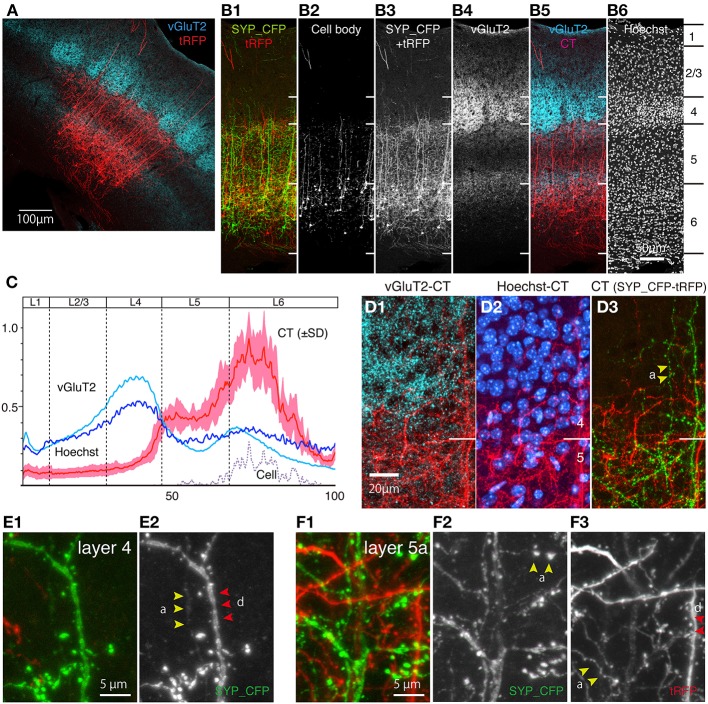
**CT cells in S1BF exhibit highly layer-specific process extension**. The intracortical processes of the same sections shown in Figure 3 were examined in reference to vGluT2 signals, which represent thalamocortical terminal boutons. All the images used here are maximal projection stacks of confocal sections. **(A)** The low power view of CT processes (tRFP: red) and vGluT2 (pseudocolored to light blue) in S1BF. Note that vGluT2 immunostaining visualizes the “barrel structures,” which are the thalamocortical terminations relaying whisker inputs. **(B)** Side-by side views of the multiple staining. The section shown in **(A)** had signals in four channels. The SYP_CFP and tRFP signals that represent the CT signals were enhanced by antibodies and shown as green and red in **B-1**. These signals were mixed and represented in gray scale in **B-3** and in red in **B-5**. Panel **B-2** shows the contrast-adjusted image of **B-3**, in which only the strong signals in the cell bodies are visible. The counterstaining of the vGluT2 antibody was detected in the far-red channel, and shown in gray scale in **B-4** and in light blue in **B-5**. The layer borders were determined on the basis of Hoechst nuclear staining (**B-6**). **(C)** The fluorescence intensity of CT (only tRFP), Hoechst, and vGluT2 signals at different lamina positions were shown as a graph. The result of seven independent samples were normalized and averaged. The x-axis represents the relative lamina positions (pia = 0, white matter = 100) and the y-axis represents the normalized fluorescence intensities. The standard deviation (SD) is shown as light red for CT signals. The plot for “cell” represents the signals for the contrast-adjusted images of the CT cells. **(D)** The layer4/5 border of panel **(A)** was magnified. The three panels represent different channels of the same section **(D-1,D-2)** The CT processes of SYP_CFP and tRFP were both pseudocolored in red. vGluT2 **(D-1)** and Hoechst **(D-2)** signals were represented by light blue and blue, respectively. Note the small gap between the vGluT2-stained thalamocortical terminations and layer 4/5 border. Layer 4/5 border here is determined on the basis of neuronal densities revealed by Hoechst staining. **(D-3)** The CT processes of SYP_CFP and tRFP were shown by green and red, respectively. **(E,F)** Magnified views of CT processes in layers 4 and 5a. SYP_CFP and tRFP signals were shown together in green and red, respectively, or were shown separately in gray scale. Yellow arrowheads denoted as “a” indicate axons and red arrowheads with “d” indicate dendrites, identified by morphology (see Section Materials and Methods for criteria). Panels **(E-1,F-1)** show SYP_CFP and tRFP signals in green and red, while **(E-2,F-2)** show only SYP_CFP signal in gray scale. Panel **(F-3)** show tRFP signals in gray scale.

As shown in Figures 3B,C, the dendritic/axonal extensions of the labeled CT cells were highly layer-specific. Compared with the Hoechst nuclear staining, it appeared that the upper limit of extension is just at the border between layers 4 and 5. To make this point clear, we performed in-depth analyses of the lamina specificity for CT cell processes, using vGluT2 as an additional marker for layer 4. As shown in Figure 4A, vGluT2 antibody heavily stains the barrel-shaped thalamocortical terminations that target layer 4 (Kaneko et al., 2002). The side-by-side comparison showed that the apical processes that originated from the CT cells in deep layers mostly stall at the border between layers 4 and 5 (Figure 4B). In addition, we observed a few thick tufted dendrites and thin axonal fibers to reach layer 1. Consistent with previous reports (Hattox and Nelson, 2007; Ueta et al., 2013), these dendrites appeared to originate from layer 5 CT cells (data not shown). While the majority of the processes were below the layer 4/5 border, there were also processes that reach the bottom of layer 3 (Figures 4B-1,B-3, see below). To quantitate the relative abundance of these processes, we measured the fluorescence intensity at various lamina positions and averaged after normalization. Figure 4C shows the summary graph of seven independent double infection experiments. As this graph shows, the lamina profile of fluorescence (tRFP) intensity for the CT processes (red plot: average and standard deviation are shown) was quite consistent despite variable transduction efficiency. We observed a clear-cut border of process extension at exactly the lower limit of vGluT2 staining and Hoechst staining.

**Figure 5:**
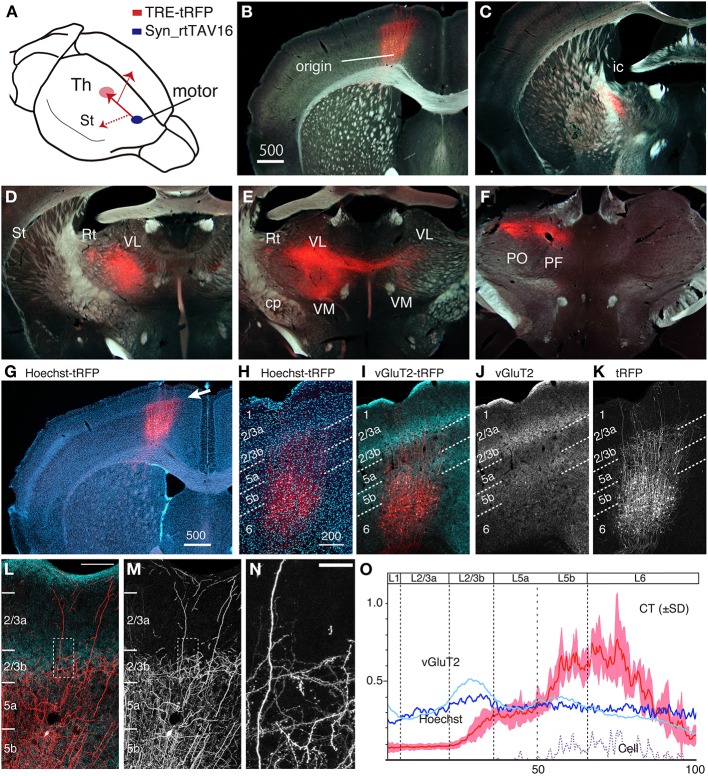
**The intracortical and extracortical projection patterns of CT cells in M1**. NeuRet vector carrying TRE-tRFP was injected into the thalamus and AAV vector carrying Syn-rtTAV16 was injected into M1. The expressed tRFP (red) was detected by immunostaining. **(A)** Schematic view of double injection. **(B–F)** The coronal images of the tRFP signals were aligned in order from anterior to posterior. The tRFP signals are overlayed on dark field images of the sections. **(G)** Same section as panel **(B)**, showing the image of Hoechst staining. The arrow indicates continuous line of dense staining, which corresponds to layer 4 outside M1. **(H–K)** Identification of precise layers for CT processes (red) by counterstaining with Hoechst (panel **H**) and vGluT2 immunostaining (panels **I** and **J**). The lamina borders denoted by the white dotted lines were determined according to the vGluT2 staining. **(L,M)** High-power view of panel **(I)** around the border between layers 2/3b and 5a. Panel **(M)** shows only the tRFP signals. Bar: 100 μm. **(N)** Dotted square in panel **(M)** is magnified to show fine dendritic and axonal branches with spines and boutons. Bar: 20 μm. The images used in **(H–N)** are maximal projection stacks of confocal sections. **(O)** The fluorescence intensity of CT (tRFP), Hoechst, and vGluT2 signals at different lamina positions were shown as a graph. The result of four independent samples were normalized and averaged. The standard deviation (SD) is shown as light red for CT signals. The plot for “cell” represents the signals for the contrast-adjusted images of the CT cells.

At first glance, our result seems to contradict the generally held view that the CT collaterals target layer 4 (see Section Discussion for more details). Actually, we did observe axons and dendrites that run vertically through layer 4, except those that reach as high as layer 1 (Figures 4B,D,E). These axons and dendrites had spines and boutons, suggesting that they form synapses in layer 4. However, they were few in numbers and lacked fine branching if present (Figure 4D-3). In contrast, the processes in layer 5 had numerous thin branches extending into various directions and forming a complex network (e.g., Figures 4D,F). These processes included both axons (yellow arrowheads, denoted as “a”) and dendrites (red arrowheads, denoted as “d”) based on their morphology. The double staining with the vGluT2 antibody suggested that CT axons and dendrites face a barrier at the border (Figures 4B,D). To be more exact, these axons and dendrites penetrate into the deepest part of cell-dense compartment of layer 4 (Figure 4D-2) but do not go further into the vGluT2-labeled thalamocortical compartment (see Figures 4B-5,D-1). From these observations, we suggest that the CT cells in mouse S1BF mainly send outputs and receive inputs in layers 5 and 6 with only minor contribution within layer 4. In addition, there appear to exist outputs and inputs in upper layers including layer 1 for layer 5 CT cells.

### CT cells in M1 have similar intracortical axonal/dendritic fields to those of S1BF

S1BF has a highly specialized lamina structure in that it receives a massive input from the thalamus, which is manifested in the dense staining of vGluT2 (Figure 4A). The layer-specific nature of the CT processes in S1BF led us to wonder whether such property is conserved across areas. To examine this point, we performed the double infection experiment for CT cells in M1. As shown in Figure 5A, the combination of the retrograde and local infection vectors are essentially the same as those tested for S1BF: the NeuRet vector was injected into ventrolateral thalamic nucleus (VL) and AAV was injected into M1. As a result, we obtained strong expression of tRFP in the motor CT cells (Figure 5B). As in the case of S1BF injection, we were able to follow their trajectory from soma toward the thalamus: we observed the CT axons to go through internal capsule (Figure 5C), innervate reticular nucleus (Rt), VL, VM (ventromedial thalamic nucleus), PO and PF (parafascicular thalamic nucleus) of thalamus (Figures 5D–F). We also observed minor collaterals to striatum and pons (data not shown). A characteristic feature of the motor CT innervation was that the CT axons branch off at VL to spread into the contralateral VL and VM (Figure 5E), consistent with the previous anterograde studies (Alloway et al., 2008). Long-distance collateral extensions of CT cells in S1BF and M1 are summarized in **Figure 9A**.

Within cortex, we observed a layer-specific pattern of process extensions of the motor CT cells (Figure 5G), which was quite similar to that in S1BF. To identify the exact layers of process extensions, we used the vGluT2 immunostaining as the primary marker. In previous studies, three distinct peaks of vGluT2 immunostaining (Figure 5J) have been defined as layers 1, 2/3b, and 5b in M1 (Morishima et al., 2011; Ueta et al., 2013). As to layer “2/3b,” the second layer of abundant vGluT2 signals correspond to layer 4 in the adjacent areas (compare Figures 5G–I). Furthermore, RORbeta mRNA, a well-known layer 4 marker, was weakly expressed in this layer (data not shown). We thus believe that layer “2/3b” in M1 is equivalent to layer 4. As to layer “5b,” the third layer of abundant vGluT2 signals correspond to layer 6 in the adjacent areas (Kaneko et al., 2002). However, we observed expression of ER81 mRNA in this layer (data not shown), which is a marker for layer 5 (Watakabe et al., 2007). Besides, FoxP2, a marker for layer 6 (Ferland et al., 2003), was weak and scattered in this layer (data not shown), supporting its identity as “layer 5.”

With this layer definition, the CT cells were mainly positioned in layers 5b and 6, while extending their processes as high as layer 2/3b (Figure 5K). The CT terminations in M1 appeared to intermingle with the vGluT2-enriched TC terminations but were still restricted around the border between layers 2/3b and 5 (Figures 5L–N). At least in four cases of independent injections, these positions were constant, as shown in Figure 5O. Compared with the CT lamina profile in S1BF, the restriction of upward extension of CT processes was more lax in M1, which is manifested in more fluorescent signals in layer 2/3b (Figure 5O). Based on these findings, we conclude that the basic interlamina architecture of the CT cells and their processes are similar across M1 and S1BF, despite various area-specific differentiations (see **Figure 9D** for summary).

### Callosally-projecting CC cells exhibit complex collateral branching patterns

**Figure 6:**
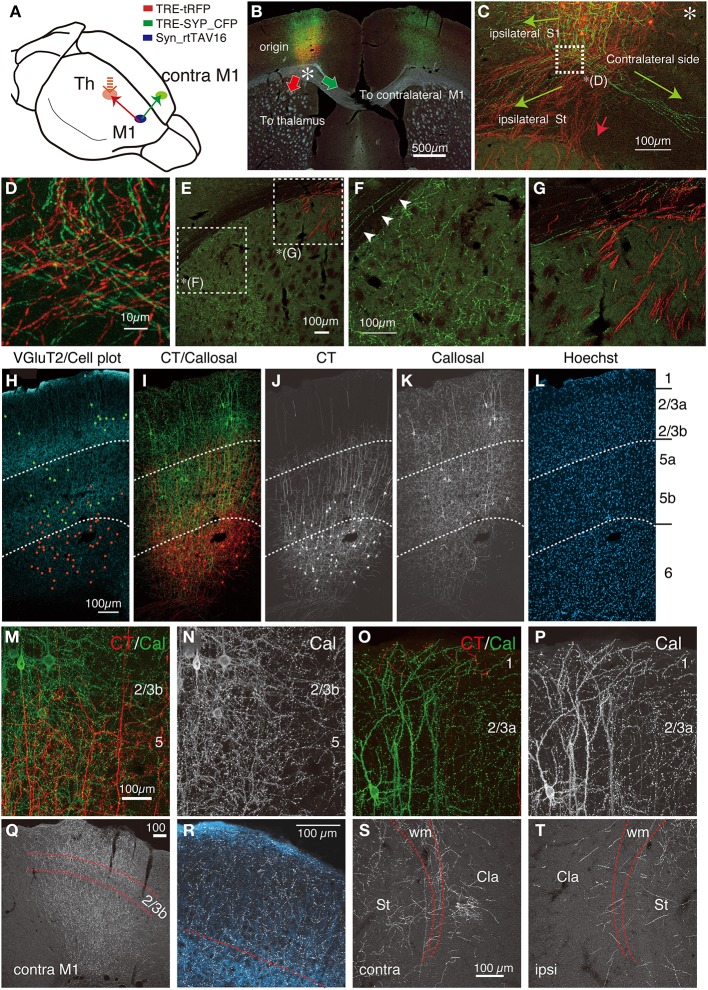
**Double labeling of CT and callosal cells in M1**. AAV vector encoding Syn-rtTAV16 was injected into M1 and two NeuRet vectors carrying TRE-tRFP and TRE-SYP_CFP were injected into the thalamus and contralateral M1, respectively. By this triple injection, CT and callosally-projecting CC cells were differentially labeled by tRFP (red) and SYP_CFP (green), respectively, which were detected by immunostaining. **(A)** Schematic view of triple injection. **(B)** The coronal section at the level of M1 showing the soma and the trajectories of CT (red arrow) and callosal CC (green arrow) cells overlayed on the dark field view of the tissue section. The asterisk indicates the split point for the two projection cell types. **(C)** The differential trajectories of axonal projections below the cortex (the asterisk in **B**). Three main projections were observed for the callosal CC cells (green). The projection toward the “ipsilateral S1” occurred intracortically. The other axons reached the white matter and split bilaterally: the medial projection extended to the “contralateral side” via the white matter and the lateral projection innervated “ipsilateral striatum (St).” The CT projections descended down to enter the striatum (red arrow). **(D)** The magnified view of the white square in panel **(C)**. Note that CT and callosal axon fibers intermingle extensively. **(E)** The magnified view of the dorsolateral part of the striatum in **(B)**. The complementary distribution of the CT and callosal axon fibers can be seen in **(F,G)**. **(F)** Magnified view of **(E)**. The collaterals of callosal CC cells are distributed widely in the striatum, while those of CT cells are absent. The arrowheads indicate the collaterals of callosal CC cells running through the white matter. These axons reach the lateral ends, where they enter the striatum. **(G)** Magnified view of **(E)**. The CT axon fibers (red) enter the striatum as bundles. **(H–L)** Side-by side view of the multiple staining. The vGluT2 staining in light blue is shown in panel **(H)**, where the cell bodies of the CT and callosal CC cells were plotted as red and green, respectively. The CT and callosal CC signals in red and green are shown in **(I–K)**, either as merged **(I)** or single-channel views. The dotted lines indicating the layer borders were determined by the distribution of vGluT2 immunostaining. **(M–P)** The magnified views of M1 around the border between layers 2/3b and 5 **(M,N)** or between layers 1 and 2/3a **(O,P)**. The granular staining for callosal CC cell collaterals (green) suggests synaptic contacts across layers. **(Q)** The callosal collaterals in the contralateral M1 in gray scale. The borders above and below layer 2/3b shown by red dotted lines were determined based on the distribution of co-stained vGluT2 signals. **(R)** Magnified view of **(Q)**. The axon collaterals of callosal cells are shown in gray scale, while the counterstained vGluT2 signals are shown in light blue. **(S,T)** The collateral projections of the callosal cells to the contralateral **(S)** and ipsilateral **(T)** claustrum. Dotted lines indicate the upper and lower limits of the white matter. Cla, claustrum; St, striatum; wm, white matter. All the images except **(B)** are maximal projection stacks of confocal sections.

It is known that the callosally-projecting corticocortical (CC) cells in M1 have complex axonal collateral branches (Veinante and Deschenes, 2003; Garcez et al., 2007; Fame et al., 2011; Sohur et al., 2014). To investigate the connectional feature of this cell type in more detail, we injected the NeuRet vector and AAV to opposite sides of M1 for double infection (Figure 6A). In this series of experiment, we also labeled the CT cells for comparison. As shown in Figure 6B, we observed robust expression of SYP_CFP in both sides of M1 (green signals) that originate from the callosal cells, as well as tRFP expression of the CT cells. The soma for the callosal cells were located mainly in layers 2–5 (Figure 6H, green dots), and extended processes from layer 1 through 5 (Figure 6K). The callosal cells were also present in layer 6 but the number was low. At higher magnification, we observed numerous small dots for SYP_CFP signals, which were considered to be spines and boutons that participate in synaptic connections (Figures 6M–P). The cell bodies of the CT cells were located in layers 5b and 6 (see above), and intermingled with those of the callosal cells in layer 5b (Figure 6H, red dots; see Figures 6H–L for differential distributions of cell bodies and axons/dendrites).

**Figure 7:**
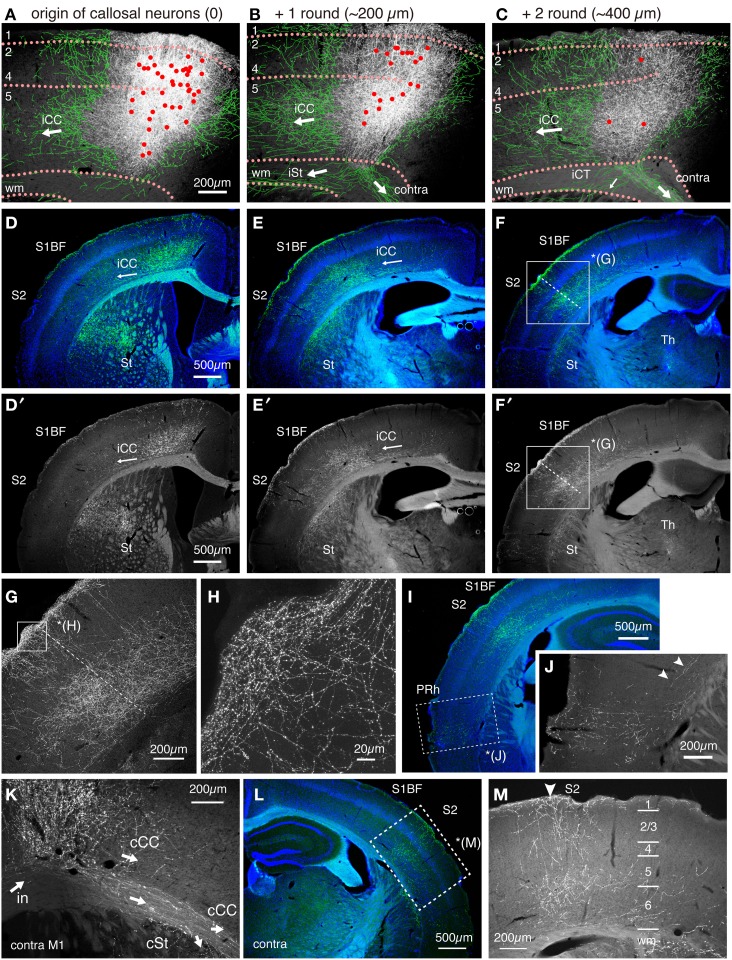
**Intracortical paths of the ipsilateral corticocortical collaterals of the callosal CC cells**. The callosal CC cells heavily innervated the ipsilateral S1BF. The trajectory of the callosal CC collaterals was followed from M1 to S1 and beyond. **(A–C)** The axonal projections around the soma in M1. The cell bodies of the callosal CC cells are plotted as red dots. To aid visualization, the thin axon fibers that originate from the injection site were manually traced in green. The orientations of the axonal fibers, as judged from the consecutive sections, are shown by white arrows. iCC, ipsilateral corticocortical projection; iCT, ipsilateral corticothalamic projection; iSt, ipsilateral corticostriatal projection; wm, white matter; contra, contralateral projection through corpus callosum. **(D–F,I)** The coronal images of the SYP_CFP signals (green) were aligned in order from anterior to posterior. The SYP_CFP signals are overlayed on Hoechst-stained images (blue). Panels **D′–F′** are the same as **D–F** but show only the SYP_CFP signals in gray scale. Note the flow of intracortical axon collaterals (iCC) from M1 to S1BF, S2, and perirhinal cortex (PRh). The boxed region in **(F,F′)** is magnified in **(G)**. The dotted lines in **(F,G)** indicate the S1/S2 border as defined by Hoechst staining. **(H)** Magnified view of **(G)** (layer 1). **(J)** Magnified image for SYP_CFP signals in the perirhinal cortex (PRh, boxed region) of **(I)**. The arrowheads indicate the intracortical collaterals that provide perirhinal terminations. **(K)** The axonal projections beyond the contralateral M1. The collaterals within the white matter proceed laterally to enter striatum (cSt) or turn upward to enter the cortical gray matter (cCC). There are also collaterals that proceed within the cortex to caudolateral areas. **(L,M)** The axonal projections to the contralateral S1 and S2. The innervation is columnar and involves layers 1 through 6. The region denoted by dotted rectangle in **(L)** is magnified in **(M)**. The arrowhead in **(M)** indicates the S1/S2 border.

The axon collaterals for the callosal CC cells and CT cells took different paths toward extracortical targets (Figure 6C). The CT axons descended straight into the white matter and the adjacent striatum to take routes into the internal capsule (Figure 6C, red arrow, Figure 6G). On the other hand, the axons of the callosal CC cells descended vertically within the cortex, split bilaterally on entering the white matter and proceeded to both ipsilateral and contralateral sides (Figures 6C,D). To the contralateral side, the axons traversed the corpus callosum and bent vertically upward just below the injection site of the retrograde vector to enter the gray matter, where they arborized extensively from layer 1 through 6, with slight reduction in layer 2/3b and 5a (Figures 6Q,R). A part of these axons further extended within the white matter (Figure 7K) to innervate the contralateral striatum as well as the claustrum (Figure 6S). Notably, the claustral innervation by the callosal CC cells was more conspicuous in the contralateral side than in the ipsilateral side (Figures 6S,T). The axons that proceeded toward the ipsilateral side, were positioned in the lower part of the white matter or beneath it (Figure 7B), which eventually innervated the ipsilateral striatum extensively (Figures 6E,F).

By principle, all the labeled “callosal” CC cells have projections to the contralateral side via white matter. These same cells also extended axon collaterals to the barrel field (S1BF), secondary somatosensory cortex (S2) and perirhinal cortex (PRh) in both hemispheres (Figures 7D–M). Interestingly, the ipsilateral cortical projections were all intracortical and never entered the white matter (Figures 7A–C). These intracortical collaterals passed through layer 6 of the adjacent areas (Figures 7D/D′) and innervated layers 1, 5, and 6 of S1BF (Figures 7E/E′) and layers 1, 2/3, 5, and 6 of the secondary somatosensory cortex (Figures 7F/F′,G). The innervation in layer 1 was especially pronounced in wide cortical areas (Figures 7D–H). Our data suggested the possibility that the “callosal CC cells,” “ipsilateral S1-projecting cells” and “perirhinal-projecting cells” are the same cells labeled from different collateral terminals.

### Ipsilateral S1-projecting CC cells in M1 have collateral projections to the same target regions as those of the callosal CC cells

**Figure 8:**
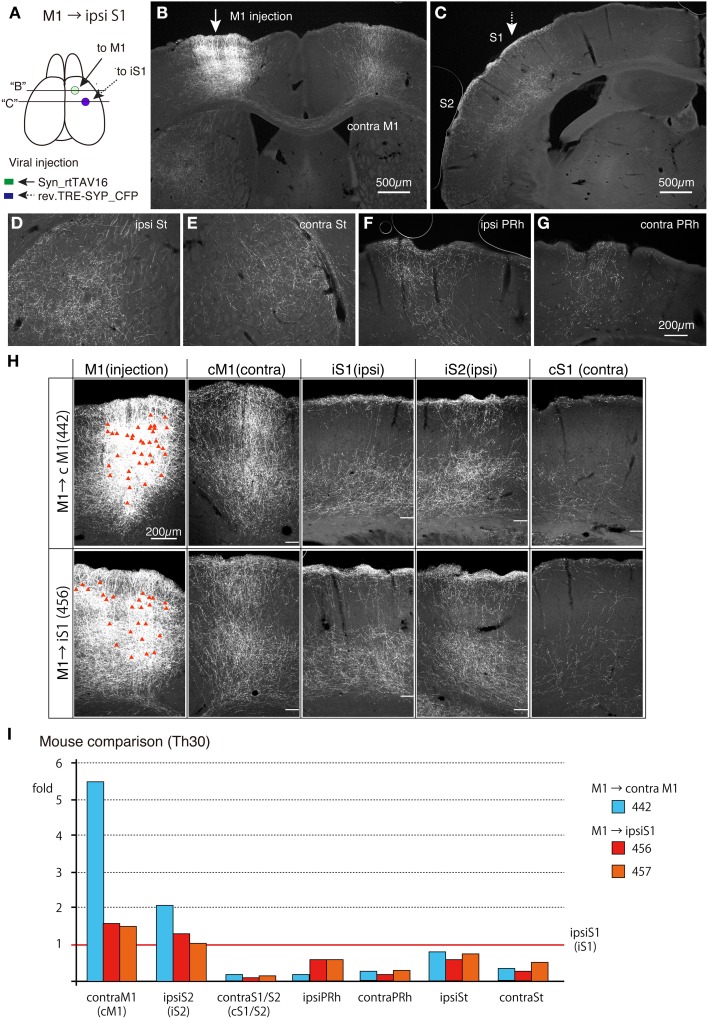
**Collateral projections of ipsilateral S1-projecting CC cells in M1 are similar to those of the callosal CC cells**. AAV vector encoding Syn-rtTAV16 was injected into M1 (shown by an arrow in **A,B**) and the NeuRet vector carrying TRE-SYP_CFP were injected into the ipsilateral S1 (shown by an broken arrow in **A,C**), and the CFP expression was detected by immunostaining. **(A)** Schematic view of double injection. The coronal planes of two injections indicated by “B” and “C” are shown in **(B,C)**. **(B)** The arrow indicates the injection site for AAV:syn-rtTAV16. Note that the projections to the contralateral M1 are all collateral projections of the ipsilateral-S1 projecting cells. **(C)** The broken arrow indicates the injection site for NeuRet:TRE-SYP_CFP. The terminal distributions shown here are essentially the same as those observed for the callosal CC cells and concentrated in layers 1, 5, and 6 across areas S1 and S2 (see Figure 7F). **(D–G)** Collateral projections to ipsilateral striatum (ipsi St), contralateral striatum (contra St), ipsilateral perirhinal cortex (ipsi PRh) and contralateral perirhinal cortex (contra PRh). These panels are at the same magnification scale. **(H)** Comparison of collateral projections of the callosal (upper panels) and ipsilateral-targeting (lower panels) cells in M1. The red triangles in the injection sites indicate the cell bodies of the labeled cells. The white bars in other panels indicate the border between the gray and white matter. Note that the lamina pattern of terminal distributions is very similar across the two types of labeling. On the other hand, the relative abundance appears to be different, which was quantified in **(I)**. In preparing these images, we combined the panels of the same mouse before adjusting the contrast, so that we can directly compare the staining intensities across different brain regions. **(I)** Abundance of collateral projections relative to those in the ipsilateral S1 was quantified (see Figure S3 for more detail). cM1, contral lateral M1; iS1, ipsilateral S1; iS2, ipsilateral S2; cS1, contralateral S1.

To test whether the callosal CC cells and ipsilateral S1-projecting CC cells exhibit the same collateral projection profiles, we injected TET-activator-encoding AAV and TRE-tRFP encoding NeuRet vector to M1 and S1 of the ipsilateral sides, respectively. As shown in Figure 8, the labeling pattern of the cells and terminals were qualitatively very similar to that of the callosal labeling. First, the infected cells in M1 were present in both upper and lower layers (Figures 8B,H; M1). Second, the callosal collaterals to the contralateral M1 innervated layers 1 through 6 with slight reduction in layers 2/3b and 5a (Figures 8B,H; cM1). Third, the widespread distribution of S1-projecting terminals concentrated in layers 1, 5 and 6 (Figures 8C,H; iS1). Fourth, sparse innervation was observed in the contralateral S1 and S2 (Figure 8H; cS1). Fifth, the striatum and perirhinal cortex on both hemispheres received collateral projections (Figures 8D–G). Finally, the claustrum on both hemispheres received projections (data not shown). It should be noted, however, that the collateral extension to the contralateral M1 of ipsilaterally-projecting CC cells (mouse #456) appeared weaker than that of contralateral-projecting CC cells (mouse #442) (Figure 8H).

To estimate the terminal distribution in more quantitative term, we measured the densities of positive pixels above threshold levels in different areas for comparison (see Section Materials and Methods and Figure S3, for details). Figure 8I demonstrates examples of such analyses for three mice. The relative terminal distributions for two ipsilaterally-labeled mice (mouse #456 and #457) were very similar. In these mice, the labeled neurons in M1 extended terminals in contralateral M1, ipsilateral S1 and ipsilateral S2 at densities with less than two fold difference, while showing only 10–20% terminals in the contralateral S1/S2. The terminal distributions of contralaterally-labeled mouse (mouse #442) were basically similar to such distribution, although contralateral M1 (cM1) received 5.5 times denser terminals than ipsilateral S1 (iS1) (Figure 8I).

## Discussion

The idea to use two viral vectors to introduce transgenes to a restricted neuronal population has been recently explored in several studies. Kinoshita et al. used essentially the same system as ours to express tetanus neurotoxin to achieve pathway-specific blockade of neurotransmission (Kinoshita et al., 2012). Xu et al. used WGA-cre AAV for retrograde transsynaptic infection and double-floxed tetanus toxin for local infection to achieve pathway specific neuronal blockade (Xu and Sudhof, 2013). Masamizu et al used retrograde infectivity of AAV9-cre to target corticospinal neurons for specific expression of floxed GCaMP in mouse M1 (Masamizu et al., 2014). The strength of these studies is that they were able to manipulate a select set of projection neurons among others. The usefulness of such double infection approach for morphological investigation, however, has yet to be fully achieved. In this regard, we have shown that our method labels neurons in their entirety including collateral branching of long-distance projections. Such enhanced imaging was made possible by using the TET-system, which helped amplify the gene expression level (Hioki et al., 2009). The Golgi-like staining that we achieved in this study may provide a breakthrough in analyzing the complex cortical wiring of non-human primates (e.g., Rockland, 2013).

Technically speaking, the key of our double infection method was the selection of the viral vector systems for transduction. The retrograde vector used in this study is an HIV vector pseudotyped with a newly developed chimeric glycoprotein of VSV and rabies virus (Kato et al., 2011c). With our preparations, we observed minimal damage around the injection site (Figures S1, S2), suggesting low toxicity of the viral vector. Other retrograde viral vectors, such as rabies (Wickersham et al., 2007), HSV (Sato and Svoboda, 2010), VSV (Beier et al., 2011), pseudorabies (Ugolini, 2010) and adenoviral vectors (Tomioka and Rockland, 2006) can be potentially used for the same purpose. In the case of rabies, the combinatorial strategy can also be used to label transsynaptic connectivity (Huang et al., 2013). However, how these vectors rival our system in terms of transduction efficiency and toxicity remains to be elucidated in further studies. The use of AAV vectors for local vectors was also a key factor for successful double labeling. Because of their physical stability, AAV could be highly purified and concentrated by ultracentrifugation, enabling efficient and low-damage local injection. In addition, AAV1 spread better than VSV-G coated lentiviral vector or AAV2 in the cortical gray matter (data not shown), which increased the possibility of double infection. Although potential tropism of AAV vector needs to be considered in evaluating the result of double infection, AAV1 is reported to have broad tropism for neuron subtypes (Wang et al., 2014).

The ability to examine a group of cells with same connectivity in Golgi-like detail is obviously a big advantage not possible in the classic anatomical methods. With our method, characteristic features of a particular projection neuron subtype could be captured in a single experiment, without the need for laborious reconstruction (e.g., see Figure 4A). On the other hand, the high density labeling as shown here is not suited for single cell tracing. We believe that further enhancement of the method should be possible by incorporating the rapidly expanding genetic tools. For example, combined use of cre-lox system may enable low-density labeling (Rotolo et al., 2008) or adding additional layer of cell type specificity (Huang and Zeng, 2013). We would also benefit from improvement in viral technology. It is currently difficult to restrict the spread of virus to a small region, while maintaining the high rate of transduction. Conversely, it is also difficult to spread viral vectors to very wide areas by a single injection. It is also a concern that AAV exhibits retrograde infection albeit at a very low efficiency (Burger et al., 2004). Manipulation of capsids for AAV (Kwon and Schaffer, 2008), glycoproteins for lentiviral vectors and/or utilization of other viral vectors can potentially solve these problems by changing the infectious property. If we could precisely target different layers for local infection, for example, we can distinguish layer 5 and 6 CT cells, which were intermixed in the current study. Finally, we point out the importance of precise mapping of the connected regions by electrophysiology or by *in vivo* fluorescent tracing (Ichinohe et al., 2012).

One limitation of the current method is that we can only analyze the connection that is already known. Our method is not the technique to map the novel connectivity. But the method to examine the extra and intracortical collateral is as yet very limited. We believe that such information should become essential in understanding primate cortex, where tens of areas are interconnected in a precise manner (Van Essen et al., 1992; Hagmann et al., 2008).

### Axonal extension and dendritic arborization of CT cells in the mouse cortex

In the visual cortices of cats, monkeys and tree shrews, the axon collaterals of layer 6 CT cells are thought to spread extensively within sublayers of layer 4 (Gilbert, 1983; Lund et al., 1994; Fitzpatrick, 1996). The collateral branching of mouse CT cells visualized in this study was quite different from such patterns. In S1BF, the axon collaterals in layer 4 extended vertically without branching, whereas those in layers 5 and 6 branched and extended in various directions (Figure 4). The dendritic arborization was also poor in layer 4, suggesting that the synaptic transmission to and from layer 6 CT cells should occur mainly in layers 5 and 6, unlike in cats or primates.

Despite a view based on cat and monkey studies that layer 6 CT cells target layer 4, the single cell tracing study in S1BF (Staiger et al., 1996; Zhang and Deschenes, 1997; Tanaka et al., 2011) or dendritic analyses in S1BF or V1 (Zarrinpar and Callaway, 2006; Tanaka et al., 2011) of rodents have also provided data that are in good agreement with our data. For example, the single cell tracing study in rat S1BF, described two cell types of CT cells, which either has presynaptic terminals across layers 4–6 in a narrow column, or has widely branched collaterals in layer 5 (Zhang and Deschenes, 1997). Because of relatively small size of sampling, however, the relative abundance of these two types had been unclear. Our data suggest that the latter cell type dominates the intrinsic innervation by the CT cells overall.

**Figure 9:**
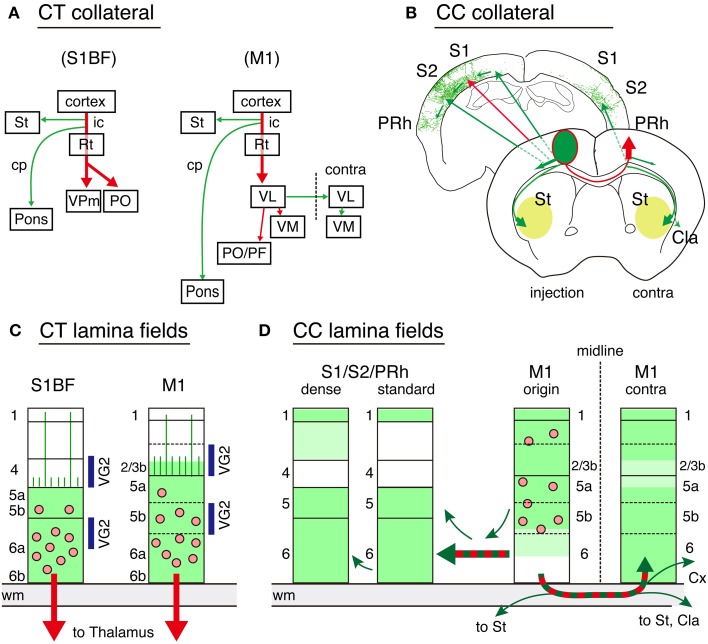
**Schematic representations of CT and callosal connectivity**. The results of the double infection data for CT and callosal cell labeling are summarized. In all the panels, the red arrows indicate the targeted pathways specified by the TET double infection, while the green arrows indicate the adjunct collaterals labeled together. **(A)** Global collateral projection patterns for CT cells in S1BF and M1 are shown. The dotted line for M1 indicates the midline. **(B)** Global collateral projection patterns for callosal cells. The two coronal planes are shown as representatives. The frontal plane represents the projection from the soma to the contralateral M1 and the trajectories for collateral branches. The corticostriatal collaterals proceed bilaterally at this plane to enter from the lateral sides of the striatum to spread anteriorly and posteriorly (pale green). The ipsilateral corticocortical projections proceed caudolateraly within the cortex through layer 6 to S1, S2, and perirhinal cortices. The posterior plane shows the manual tracing of Figure 7F as a representative trajectory. **(C)** The local spread of processes (including both axons and dendrites) for CT cells is shown by green color. The pink dots represent the cell bodies. Layers 1 through 4 are mostly devoid of CT processes except the dendrites and axons that reach layer 1, which stem from layer 5 CT cells, or those that span layer 4 with few branching. These vertical processes are represented as vertical lines in panel **(C)**. The layers with abundant thalamocortical terminations (VG2) are indicated by blue bars on the sides. **(D)** The local spread of processes for callosal cells in M1 (M1 origin) as well as the axonal terminations in the remote cortex are shown by green color. We observed two types of innervations in S1. In the dense region, we observed vertical extensions of the collaterals in layers 2 and 3, whereas a wide area of other S1 (standard) had collateral branches in various directions in layers 1, 5, and 6. Cla, claustrum; Cx, cortex; cp, cerebral peduncle; ic, internal capsule; PRh, perirhinal cortex; Rt, reticular thalamic nucleus; St, striatum; VL, ventrolateral thalamic nucleus; VM, ventromedial thalamic nucleus; VPm, ventral posteromedial thalamic nucleus; PF, parafascicular thalamic nucleus; PO, posterior thalamic nuclear group.

The distribution patterns of CT terminals observed in this study are consistent with recent studies employing Ntsr-1 cre mouse line (Lee et al., 2012; Olsen et al., 2012; Bortone et al., 2014). In this series of studies, it was shown that Ntsr1-cre is expressed specifically in CT cells and these cells specifically recruit the inhibitory cells in deep layers in mouse V1. Furthermore, a careful analysis of laminar target for Ntsr1-cre line suggested that CT cells mainly target layer 5a but not 4 in S1BF and V1 (Kim et al., 2014). Finally, laminar analyses of excitatory local circuit by glutamate uncaging revealed that output from layer 6 cells is mainly targeted at layers 5 and 6, but not above layer 4 in M1, S1, or S2 (Hooks et al., 2011). Thus, the layer-specific intracortical connectivity of CT cells appears to be overall conserved across areas in rodents (Figure 9C), but not necessarily so in cats or primates.

### Characterization of CC neurons by long-range collateral projection patterns

To our knowledge, it had not been known that perirhinal-projecting neurons in M1 have collateral to the contralateral M1. Nor had it been known that the callosal cells have collateral projection to the contralateral claustrum. These findings extend our concept of “callosal” CC neurons to include these projection cell types in addition to “S1-projecting” and “corticostriatal” neurons, which are also shown to have callosal collaterals by us and by others (see Figure 9B, Veinante and Deschenes, 2003; Mitchell and Macklis, 2005; Sohur et al., 2014). Obviously, these examples raise the question of what should be considered as the “cell type,” because the same cells are classified differently depending on which collaterals you pay attention to Watakabe et al. (2007), Greig et al. (2013), and Packer et al. (2013). Regarding this point, we propose a hierarchical classification scheme based on developmental history.

Recent studies revealed that the initial step of molecular genetic program specifies cortical pyramidal cells into either “subcerebral projecting neurons” in deep layers or “callosal projecting neurons” in upper layers (Molyneaux et al., 2007; Britanova et al., 2008; Leone et al., 2008; Fame et al., 2011). These two cell classes exhibit differential axon outgrowth from the beginning (Koester and O'Leary, 1993). The final connectivity phenotypes of cortical neurons are then determined through genetic and activity-dependent control of axon outgrowth and elimination (Luo and O'Leary, 2005; Mitchell and Macklis, 2005; Greig et al., 2013). In this developmental context, it is reasonable to group “callosal” cells as one cell class that comprises cells with various long-distance targets. Included in this cell class are CC neurons projecting to contralateral and ipsilateral sides (including perirhinal-projecting neurons), bilaterally-projecting corticostriatal cells (IT-type; Wilson, 1986; Reiner et al., 2010) and corticoclaustral cells. Not included in this cell class are CT cells, corticopontine cells, and ipsilateral-projecting corticostriatal cells (PT-type; Levesque et al., 1996; Reiner et al., 2010), which send main axons into the internal capsule. This classification scheme is consistent with class I/II classification of layer 5 neurons (Kasper et al., 1994; Molnar and Cheung, 2006; Morishima and Kawaguchi, 2006). It is intriguing that callossally and ipsilaterally-projecting CC cells in M1 exhibited very similar collateral extension patterns.

On the other hand, our analyses also suggested the heterogeneity of CC cells in terms of relative abundance of the collateral branches. The double labeling studies of retrograde tracers also suggests partial overlaps between corticostriatal and corticocortical populations (Mitchell and Macklis, 2005; Sohur et al., 2014) or between corticostriatal and perirhinal-projecting populations (Hirai et al., 2012). At this point, we are not certain whether we should consider such heterogeneity as reflecting the phenotypic variations of a single cell type or as the diversity of cell types itself. Either way, it should become important to classify cell types based on overall collateral projection patterns rather than on just by one of the targets.

Apart from the classification issue, we think it intriguing that the ipsilateral cortical projections of the callosal cells proceed intracortically, while the callosal and corticostriatal collaterals proceed via the white matter. The conduction velocities of axonal pathways are greatly influenced by myelination and other parameters (Chomiak et al., 2008; Kimura and Itami, 2009; Seidl, 2013). The differential routing may be important for interlinking multiple targets with specific timing differences or delays (Rockland, 2013). In this regard, the corticocortical projections in monkeys generally take the white matter route (Schmahmann and Pandya, 2009). Such difference may contribute to the primate-specific organization of long-range cortical circuits.

### Conflict of interest statement

The authors declare that the research was conducted in the absence of any commercial or financial relationships that could be construed as a potential conflict of interest.
