# Supplementary material for: Simultaneous visualization of extrinsic and intrinsic axon collaterals in Golgi-like detail for mouse corticothalamic and corticocortical cells: a double viral infection method
Source: Front Neural Circuits. 2014 Sep 17;8:110. doi: 10.3389/fncir.2014.00110 (PMC4166322; doi:10.3389/fncir.2014.00110)
Supplement: Supplementary file 1 [file DataSheet1.PDF]

Supplementary figures

Fig. S1 Background of AAV-TRE vector

In this series of experiments, we injected into S1BF either AAV: TRE_hrGFP alone (panles A-D: TRE vector only) or in combination with NeuRet: MSCV-tTA into the thalamus (panels E-G: TET double infection). The tissue sections were immunostained with anti-hrGFP antibody (green; Vitality hrGFP antibody, Agilent Technologies Inc.) and with NeuN antibody (red; MAB377, Millipore). So that we can directly compare the strength of signals of the two samples, immunostaining was performed side by side and the photos were taken and the contrast was adjusted in exactly the same way (for panels B and F as well as for C and G). Note weak expression of hrGFP for cell body and dendrites for "TRE vector only". (A) A merged view for hrGFP and NeuN immunostaining of TRE-vector only sample. (B) hrGFP signals alone of panel A. (C) magnified view of panel B denoted by white dotted square. (D) ISH of hrGFP sequence, which visualized the injected AAV genome (A.W., unpublished data). The arrow indicates the site of injection. This experiment assured that the injection occurred successfully. (E-G) A merged view and single channel views for hrGFP and NeuN immunostaining of TET double infection sample. The dotted squares are magnified in panels H-L (H) A magnified view of panel F. Compare this panel with panel C. These panels were processed in exactly the same way. (I-N) Damages to the tissue were estimated by lack of NeuN immunostaining for the TET double infection sample shown in panels E-G. The dotted white boxes were magnified in panels I-L. (I and J) The center of the injection exhibited lack of NeuN signals in the cell bodies which accompanied the loss of transgene signals. (K and L) The infected cells adjacent to the injection site showed no sign of cell damage. (M and L) A magnified view of panels K and L, showing strong NeuN signals for hrGFP-positive neurons.

Fig. S2 Terminal morphology of infected neurons around the injection site.

Panel J of Fig. 2 was magnified to show that fine morphology of terminal boutons can be observed even around the injection site. (A) and (C) Merged views of tRFP signals (red) and CTB-Alexa488. (B) and (D) Only tRFP signals are shown for panels (A)

and (C), respectively. Note dense innervation even around the center of CTB deposits. The white boxes in panels (A) and (B) are magnified in panels C and D, respectively. The arrows in panels C and D indicate potential tissue damage due to penetration of injection needles. The white box in panel D was magnified in panel E. (E) Magnified view of the injection site. The arrowheads indicate large boutons, characteristic of layer 5 CT cells. The white box is magnified in panel F. (F) Note the presence of many fine boutons and fibers.

Fig. S3 Quantitation of collateral fibers

(A) Contrast-adjusted image of contralateral M1 of mouse 442. The white dotted box indicates the region of interest (ROI) for measurement of terminal distributions. (B) The raw image of the fluorescent signals were converted to an 8 bit grey image and binarized at different threshold values. The ratio of the positive areas against the total area for the designated ROI (%area) was measured as an index of signal intensities, which is indicated below each panel. The boxes in the panels are magnified in panel C to show the detail of the binary images. As these panels show, the ratio of the positive signals changed dramatically depending on the threshold value. (D) The quantification of the terminal distribution (%area) of SYP-CFP signals of mouse#442 in various cortical areas at different threshold values (Th10~50). cM1; contralateral M1, iS1; ipsilateral S1, iS2; ipsilateral S2, cS1; contralateral S1, iPRh; ipsilateral perirhinal cortex, cPRh; contralateral perirhinal cortex, iSt; ipsilateral striatum, and cSt; contralateral striatum. Note that the shape of the graphs are similar irrespective of the threshold, (except TH10), despite large differences in the absolute values. The threshold for TH10 is so close to the background that the “positive” signals are saturated in some areas. (E) The relative intensities of terminal distribution of mouse #442 in various brain regions were normalized against the ipsilateral S1 (iS1). The relative intensities somewhat differed depending on the threshold values for iPRh and cPRh, but were quite constant for other areas.

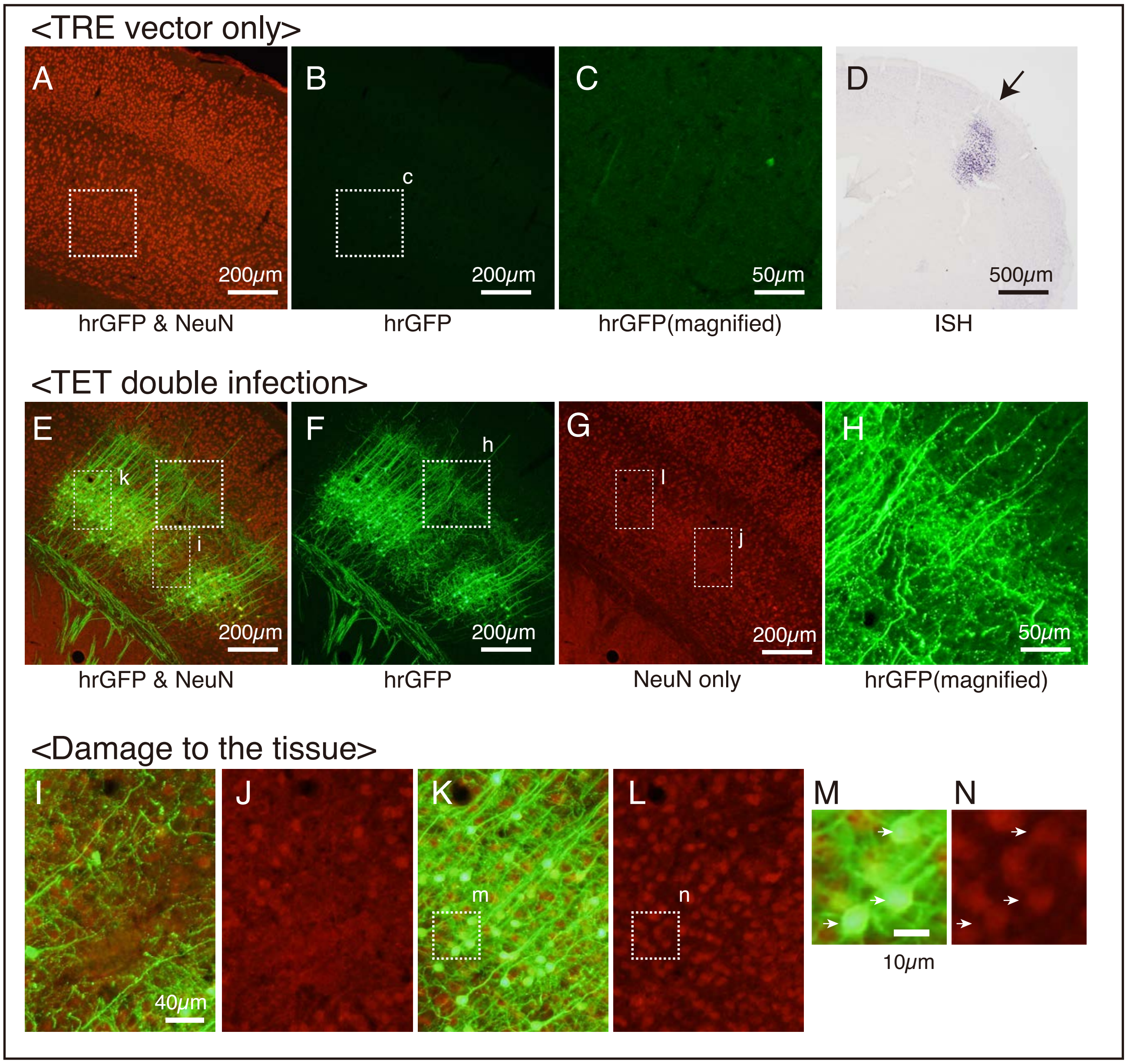

<TRE vector only>
A
B
C
D
c
200μm
200μm
50μm
500μm
hrGFP & NeuN
hrGFP
hrGFP(magnified)
ISH
<TET double infection>
E
F
G
H
k
i
h
l
j
200μm
200μm
200μm
50μm
hrGFP & NeuN
hrGFP
NeuN only
hrGFP(magnified)
<Damage to the tissue>
I
J
K
L
M
N
m
n
40μm
10μm

FigS1

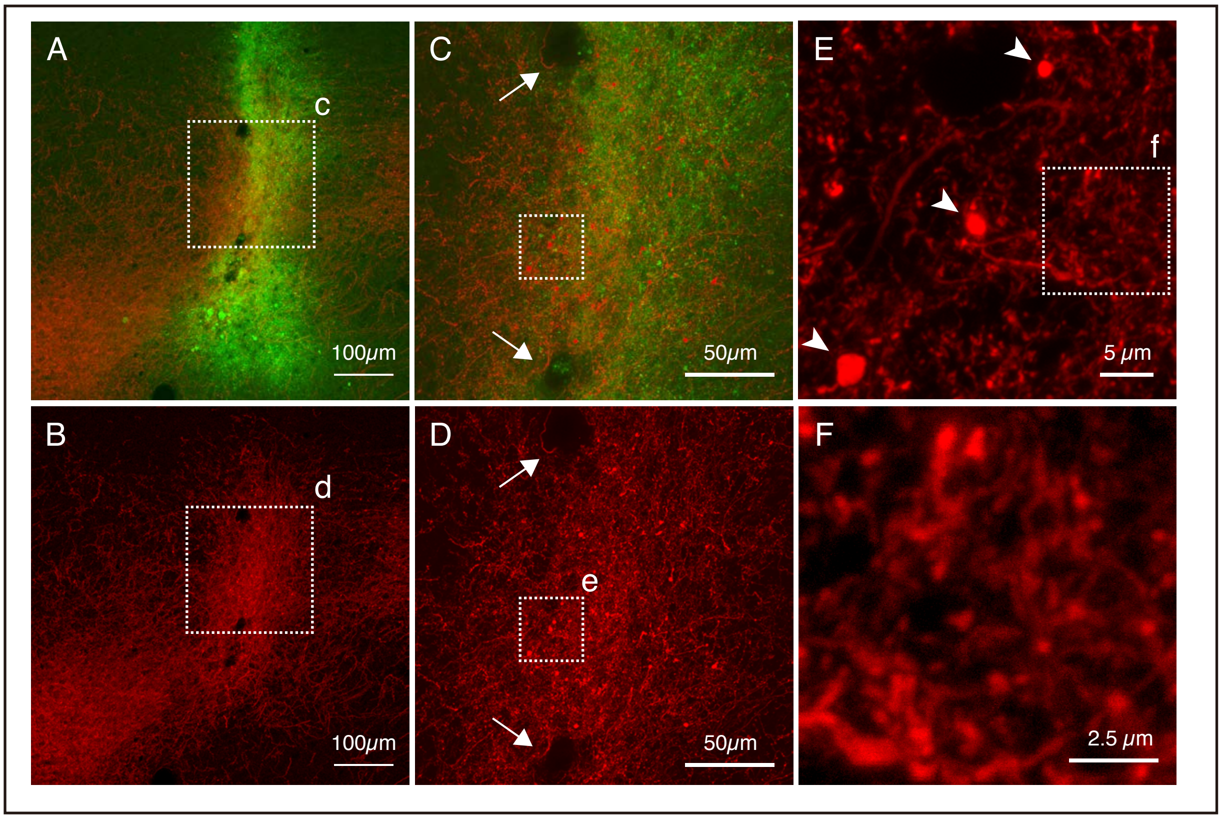

Fig. S2

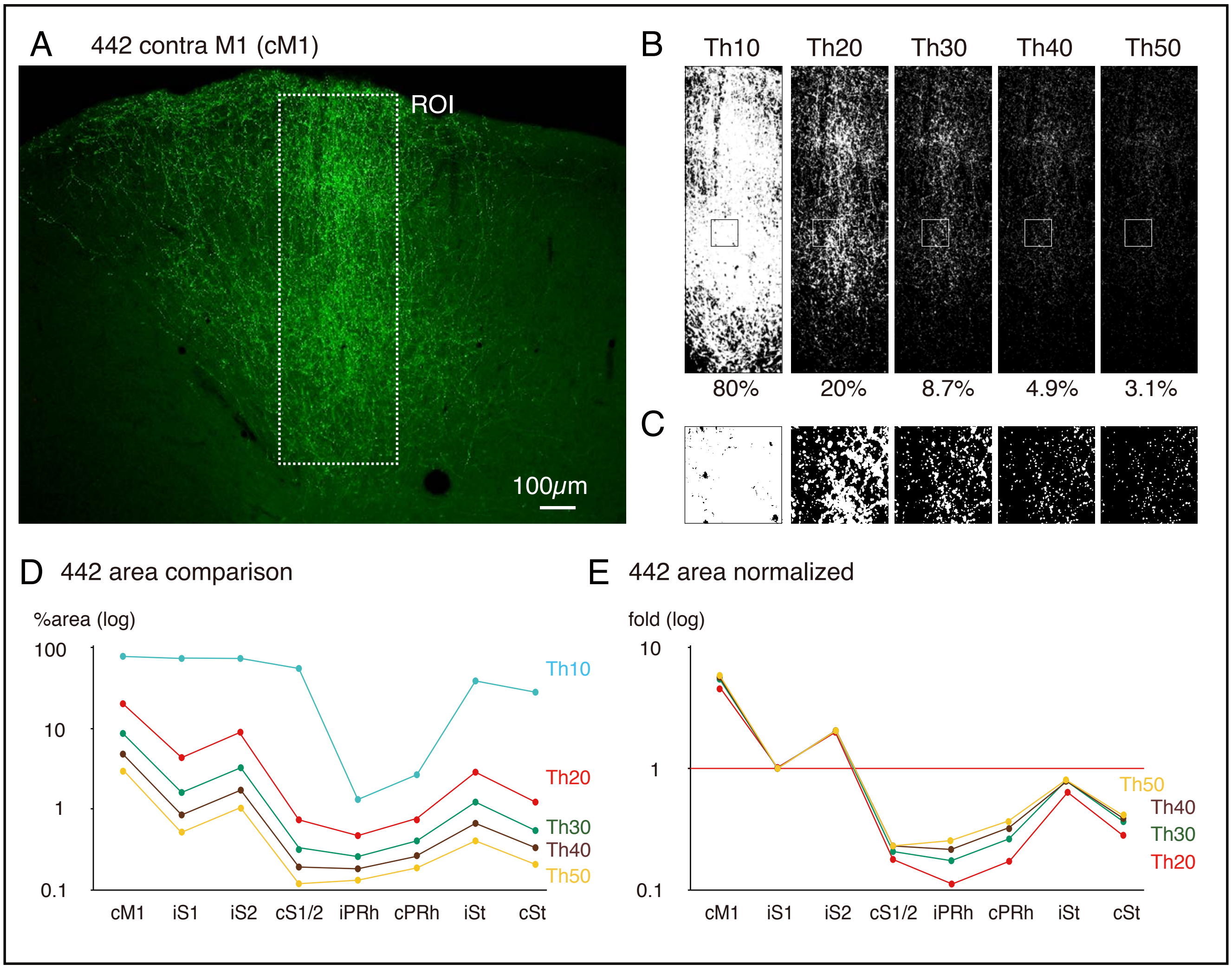

A
442 contra M1 (cM1)
ROI
100μm
B
Th10
Th20
Th30
Th40
Th50
80%
20%
8.7%
4.9%
3.1%
C
D
442 area comparison
%area (log)
100
10
1
0.1
Th10
Th20
Th30
Th40
Th50
cM1
iS1
iS2
cS1/2
iPRh
cPRh
iSt
cSt
E
442 area normalized
fold (log)
10
1
0.1
Th50
Th40
Th30
Th20

Fig. S3

Table S1

		Microscope	Objective	filter cube	pinhole size (μm)	scan	z drive (μm)
Fig.2	B	BX51	4x	G+R			
	C	Nikon A1	20x	CH2+3	25.5	1	0
	D	BX51	4x	R			
	E	BX51	4x	R			
	F	BX51	4x	R			
	G	BX51	4x	R			
	H	BX51	4x	R			
	I	BX51	4x	G+R			
	J	Nikon A1	60x(DZ6x)	CH3	28.3	5	12
	K	Nikon A1	20x	CH3	20.1	16	12.76
	L	Nikon A1	60x	CH3	38.1	55	22.08
	M	Nikon A1	20x	CH3	38.1	50	20.1
	N	BX51	4x	R			
	O	Nikon A1	40x	CH3	25.5	63	18.62
	Oi	Nikon A1	40x(DZ6x)	CH3	25.5	29	8.42
Fig.3	B	BX51	4x	B+G+R			
	C	BX51	4x	G			
	D	Nikon A1	20x	CH3	20.1	5	20.05
	E	Nikon A1	20x	CH2	20.1	5	20.05
	F	Nikon A1	60x	CH2+3	38.1	28	10.12
	G	Nikon A1	60x	CH2+3	38.1	28	10.12
	H	BX51	4x	G+R			
	I	Nikon A1	60x	CH2+3	38.1	50	18.4
	J	Nikon A1	60x	CH2+3	38.1	58	21.4
	K	Nikon A1	60x(DZ6x)	CH2+3	38.1	39	0.75
	L	Nikon A1	60x(DZ6x)	CH2+3	38.1	41	10
Fig. 4	A	Nikon A1	10x	CH3+4	32.1	3	11.18
	B1	Nikon A1	10x	CH2+3	32.1	3	11.18
	B2	Nikon A1	10x	CH(2+3)	32.1	3	11.18
	B3	Nikon A1	10x	CH(2+3)	32.1	3	11.18
	B4	Nikon A1	10x	CH4	32.1	3	11.18
	B5	Nikon A1	10x	CH(2+3)+4	32.1	3	11.18
	B6	Nikon A1	10x	CH1	32.1	3	11.18
	D1	Nikon A1	60x	CH(2+3)+4	84.6	42	8.53
	D2	Nikon A1	60x	CH1+(2+3)	84.6	42	8.53
	D3	Nikon A1	60x	CH2+3	84.6	42	8.53
	E1	Nikon A1	60x(DZ6x)	CH2+3	43.4	28	8.1
	E2	Nikon A1	60x(DZ6x)	CH2	43.4	28	8.1
	F1	Nikon A1	60x(DZ6x)	CH2+3	38.1	40	7.48
	F2	Nikon A1	60x(DZ6x)	CH2	38.1	40	7.48
	F3	Nikon A1	60x(DZ6x)	CH3	38.1	40	7.48
Fig.5	B	BX51	4x	R+W			
	C	BX51	4x	R+W			
	D	BX51	4x	R+W			
	E	BX51	4x	R+W			
	F	BX51	4x	R+W			
	G	BX51	4x	B+R			
	H	Nikon A1	10x	CH1+3	28.3	3	10.45
	I	Nikon A1	10x	CH3+4	28.3	3	10.45
	J	Nikon A1	10x	CH4	28.3	3	10.45
	K	Nikon A1	10x	CH3	28.3	3	10.45
	L	Nikon A1	20x	CH3+4	22.2	7	8.27

	M	Nikon A1	20x	CH3	22.2	7	8.27
	N	Nikon A1	20x	CH3	43.4	28	11.92

Fig.6	B	BX51	4x	G+R+W			
	C	Nikon A1	20x	CH2+3	47.5	6	7.5
	D	Nikon A1	60x(DZ3x)	CH2+3	38.1	28	10.57
	E	Nikon A1	10x	CH2+3	32.1	3	11.08
	F	Nikon A1	10x	CH2+3	32.1	3	11.08
	G	Nikon A1	10x	CH2+3	32.1	3	11.08
	H	Nikon A1	10x	CH4	32.1	3	11.15
	I	Nikon A1	10x	CH2+3	32.1	3	11.15
	J	Nikon A1	10x	CH3	32.1	3	11.15
	K	Nikon A1	10x	CH2	32.1	3	11.15
	L	Nikon A1	10x	CH1	32.1	3	11.15
	M	Nikon A1	60x	CH2+3	43.4	17	7.05
	N	Nikon A1	60x	CH2+3	43.4	17	7.05
	O	Nikon A1	60x	CH2+3	43.4	27	8.7
	P	Nikon A1	60x	CH2	43.4	27	8.7
	Q	Nikon A1	20x	CH2	38.7	9	8
	R	Nikon A1	20x	CH2+4	38.7	9	8
	S	Nikon A1	20x	CH2	26.1	13	12
	T	Nikon A1	20x	CH2	26.1	17	16.03

Fig. 7	A	BX51	10x				
	B	BX51	10x				
	C	BX51	10x				
	D	BX51	4x				
	E	BX51	4x				
	F	BX51	4x				
	G	Nikon A1	10x	CH2	24.3	9	26.25
	H	Nikon A1	60x	CH2	56.1	58	19.96
	I	BX51	4x				
	K	BX51	10x				
	L	BX51	10x				
	M	BX51	4x				
	N	BX51	10x				

Fig8	B	BX51	4x
	C	BX51	4x
	D	BX51	10x
	E	BX51	10x
	F	BX51	10x
	G	BX51	10x
	H	BX51	10x

DZ: degital zoom

CH2+3: CH2 and CH3 images merged with different colors

CH(2+3): CH2 and CH3 images merged with the same color

Specifications for Olympus BX51 fluoresence microscopy

<Objective>

4x	UPlanFI 4x/0.13
10x	UPlanApo 10x/0.40

<filter cube>

B (UV)	U-MWU2
G (green)	U-MNIBA2
R (red)	U-MWIG2

Specifications for NikonA1 confocal microscopy

Setting for multiple channel retrieval		
4CH	laser	filter
CH1(blue)	405	450/50
CH2(green)	488	525/50
CH3(red)	561	595/50
CH4(far red)	640	-
CH4>CH3>CH2>CH1		

<Objective>

10x	Plan Apo 10x
20x	Plan Apo VC 20x DIC N2
40x	Apo LWD 40x WI λS DIC N2
60x	Plan Apo VC 60x WI DIC N2

<First Dichroic Mirror>	405/488/561/640
Reflection band	400-413/487-489/560-562/636-641
Transmission band	423-478/498-550/572-626/651-750
